# Editorial: Reviews in: regulatory science

**DOI:** 10.3389/fmed.2023.1206753

**Published:** 2023-05-16

**Authors:** Sandor Kerpel-Fronius, Violeta Stoyanova-Beninska, Viviana Giannuzzi, Zhonghua Sun

**Affiliations:** ^1^Department of Pharmacology and Pharmacotherapy, Semmelweis University, Budapest, Hungary; ^2^Medicines Evaluation Board, Utrecht, Netherlands; ^3^Fondazione per la Ricerca Farmacologica Gianni Benzi Onlus, Valenzano, Italy; ^4^Discipline of Medical Radiation Science, Curtin Medical School, Curtin University, Perth, WA, Australia

**Keywords:** regulatory science, medicines development, nucleic acid-based drugs, herbal medicines, drug safety, drug pricing

Frontiers in Medicine introduced the idea of bundling several papers dealing with similar or related problems into so-called Research Topics. It is expected that this editorial concept might convey an organized, inter-linked overview of related research results to the readers. This concept provides additionally the possibility to make functional connections between research projects which are anchored in different scientific disciplines. Such combined presentation helps to broaden the scientific horizon of experts working in different fields, and supports the interpretation of their results in wider scientific and social context. This special collection has published eight manuscripts of researchers from different countries and continents reporting examples of the latest knowledge in regulatory science. The goal of our selection was to cover a broad variety of regulatory challenges emerging in connection with the development of various nucleic acid-based drugs, oncological agents, the evidence-based evaluation of herbal medicinal products, the impact of pharmacogenetic programs on healthcare and finally linking marketing approval to the speed of pricing and reimbursement decisions.

Chiu et al. wrote an extensive overview of the tasks and activities of the U.S. Food and Drug Administration (FDA) Division of Applied Regulatory Science (DARS). This division consists of interdisciplinary teams developing primarily modern biological methods for improving *in vitro* assessment of drugs effects. The publication describes several examples of new types of assays supporting regulatory decision making.

Chisholm and Critchley from Australia argued that the rapid development of artificial Intelligence (AI) and machine learning techniques will dramatically influence the future work of regulatory experts. According to their review, it is mandatory to prepare the personnel to use efficiently and critically these possibilities for leading to successful international cooperation of regulatory agencies in adapting their work to the changing scientific environment, geopolitical shifts, pandemics, shortage of raw materials and interruptions of supply chains.

McDermott et al. called attention to future importance of large scale pre-emptive panel genetic testing of many individuals for common pharmacogenetic variants underlying diseases. This genetic information could be stored in medical records and used if needed to select individualized targeted therapy for such diseases. Lack of knowledge, and the cost of the intervention were found to be the main barriers for implementing this program. Early leadership engagement, positive institutional culture, engaging stakeholders, and the selection of clinical champions were considered as facilitators to implement such pharmacogenetic service.

The intriguing problems of the regulatory classification of the rapidly enlarging group of RNA drugs with quite different biological mechanisms of action was discussed by Guerriaud and Kohli. They flagged some of the currently recognized disparities of categorizing similar products into different categories because of their different origins. In addition, the regulatory status of RNA drugs is differently defined by the EMA and FDA which obviously makes the international registration strategy difficult. The authors suggested some proposals for improving future classification based on updated definitions and recommended steps towards an international harmonization.

Over many centuries, China developed a rich collection of herbal medicines jointly referred to as Traditional Chinese Medicines. They were traditionally evaluated only empirically. Zhou et al. described a recently initiated new program that used modern comparative clinical trial methodology to provide solid scientific background for characterization of efficacy and safety. Most of the phase II and III trials are prospective, double blind, randomized, parallel group trials. The results of these trials are expected to improve both the regulatory management and evidence-based use of these products in the clinical practice. According to the authors, the number of modern clinical trials is still small compared to the great wealth of the empirical knowledge.

The optimal use of herbal medicines depends on their standardization. Following the legalization of the cannabis market, many products with different amounts of active ingredients flooded the market. In addition, the intensity of the pharmacological effects and tolerance development are individually very different. Especially for effective patient care adjusting cannabis administration according to the needs of the patients is very important. Ilan describes an administration approach called “digital medical cannabis” which is based on the 2nd generation AI system able to modify the dose for optimizing individually patient benefit.

Zhang et al. reported a systematic review comparing the time for oncologic drug approval following multi-regional clinical trials (MRCTs) as compared with single-country studies. MRCTs involving US, Europe and Japan lead to the shortest time for the approval of new oncological agents. The inclusion of additional regions prolonged the time for approval. Since bridging country trials need the least time, the authors recommend that additional single-country bridging studies be performed to shorten drug approval time if MRCTs do not apply.

The time needed for pricing and reimbursement of drugs are influenced by the observed effect differences between the new and the available therapies, the clinical importance of the new agents as well as by the scientific quality of the clinical studies. Gallo et al. analyzed the time needed for pricing and reimbursement decisions between 2018 and 2020 in Italy. They argue that the more than double time needed for decision making in case of new drugs is almost entirely due to the much longer health technology assessment procedure and related price negotiations.

We hope that combining these articles dealing with various aspects of drug development spanning from the bench to the patients, from the clinical studies through the regulatory decision and health technology assessment to the broad healthcare application will support the work of many colleagues active at various points of this complex process. Joint publication of these papers demonstrates that the decisions made at the different steps by various experts must consider the complexity of the entire process including also their social and ethical impacts.

## Author contributions

The draft and final version were prepared by SK-F. All authors contributed to the article and approved the submitted version.

